# Redefining the Minimal Editable Unit of Supramolecular Synthons: A 1→2 Lattice‐Editing Strategy in Ternary Multicomponent Crystals

**DOI:** 10.1002/advs.77573

**Published:** 2026-09-03

**Authors:** Zheng Lin, Honglei Xia, Min Li, Ruihui Wang, Ying Wang, Xiaoyu Feng, Mingjing Tang, Yuan Zhang, Qinghua Zhang

**Affiliations:** ^1^ School of Astronautics Northwestern Polytechnical University Xi'an Shaanxi China; ^2^ National Key Laboratory of Solid Propulsion Northwestern Polytechnical University Xi'an Shaanxi China; ^3^ School of Chemistry and Chemical Engineering Northwestern Polytechnical University Xi'an Shaanxi China

**Keywords:** lattice editing, minimal editable unit, multicomponent crystals, non‐classical bioisosteres, solid propellant

## Abstract

Conventional multicomponent crystal engineering is largely restricted to one‐to‐one component substitution, which often provides limited tunability or disrupts the parent lattice structure. Inspired by skeletal editing in molecular chemistry, we herein establish a lattice‐editing strategy for supramolecular crystals. This work extends the conventional understanding of the minimal editable structural unit in supramolecular crystals from individual components to local supramolecular structural units, thereby moving beyond strict numerical equivalence and enabling non‐stoichiometric component substitution while preserving the original interaction topology. As a proof of concept, a 1→2 component substitution was achieved in the ternary ionic crystal ethylenediamine diperchlorate hemihydrate (EP·0.5H_2_O) by replacing the [H_2_eda]^2+^–H_2_O composite unit with [H_2_pda]^2+^. The resulting anhydrous energetic crystal PEP fully retains the parent crystal framework, space group, cell parameters, and hydrogen‐bonding network. PEP exhibits a high density of 1.85 g cm^−^^3^, a phase‐transition temperature increased by approximately 90°C, and a 127% enhancement in formation enthalpy. In solid propellant formulations, PEP delivers higher specific impulses than AP and ADN while simultaneously improving thermal stability, environmental stability, and mechanical safety. This work demonstrates precise lattice editing in multicomponent crystals and provides a general strategy for the rational design of high‐performance functional crystalline materials.

## Graphical abstract

Inspired by skeletal editing, we propose a lattice‐editing strategy that extends the minimal editable unit in supramolecular crystals from individual components to local supramolecular structural units, enabling non‐stoichiometric one‐to‐many substitution while preserving the parent lattice architecture. The resulting energetic crystal PEP exhibits an exceptional *I*_sp_, improved thermal stability, anti‐hygroscopicity, and mechanical safety, showing great potential in high‐performance solid propellant.

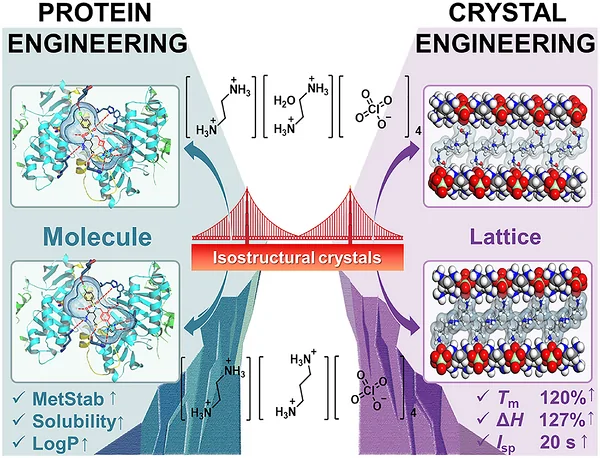

## 1 Introduction

**SCHEME 1:**
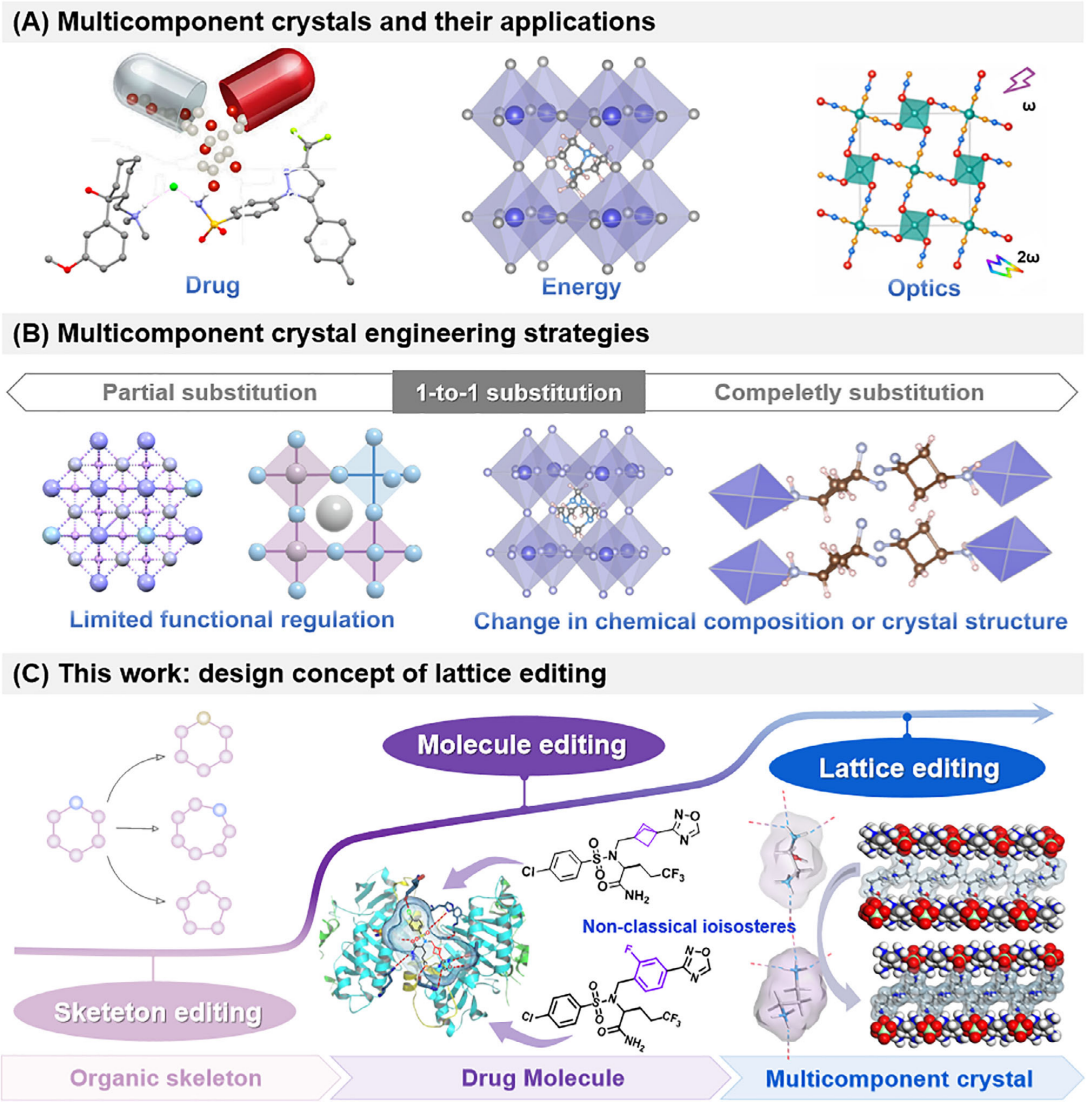
From conventional multicomponent crystal engineering to the lattice‐editing paradigm inspired by non‐classical bioisosteres. (A) Multicomponent crystals and their applications. (B) Multicomponent crystal engineering strategies. (C) Lattice‐editing strategy inspired by non‐classical bioisosteres.

Multicomponent crystals have demonstrated significant potential in diverse technological fields, including biopharmaceuticals [1], optoelectronics [2, 3], and energy applications (Scheme  1A) [4]. These materials are defined as ordered solid‑state materials that arise from the supramolecular self‑assembly of two or more chemically distinct building blocks, forming long‑range periodic lattice structures [5, 6, 7, 8]. The functional properties of multicomponent crystals are intrinsically governed by their crystal structures [9]. Consequently, precise control over structural features such as intermolecular interactions, electronic state distributions, and spatial packing motifs is essential for optimizing performance and unlocking the full technological potential [10, 11, 12].

However, traditional multicomponent crystal engineering is trapped in the bottleneck of one‐to‐one (1→1) component substitution (Scheme 1B) [13, 14]. Partial substitution (e.g., doping) can only achieve limited property tuning, while complete 1→1 substitution often causes severe lattice distortion or even structural collapse due to geometric mismatch and electrostatic incompatibility between guest and host units [15, 16, 17, 18, 19, 20, 21, 22, 23]. Worse still, such rigid substitution strategies often neglect the conservation of the local supramolecular environment. Crystal structure prediction (CSP), which aims to identify the most stable crystal structures from molecular compositions prior to experimental synthesis, becomes increasingly challenging when substitution‐induced changes generate diverse packing arrangements and complex energy landscapes. Therefore, maintaining local supramolecular environments rather than merely pursuing numerical equivalence may provide a more rational pathway for multicomponent crystal design [24, 25]. However, conventional one‐to‐one substitution lacks the flexibility to maintain local supramolecular motifs during component replacement [26]; the one‐to‐many component substitution that can retain the parent lattice has yet to be achieved, which severely restricts the development of precise post‐synthetic modification of multicomponent crystals.

Recent advances in synthetic chemistry offer valuable conceptual guidance for multicomponent crystal engineering [27, 28]. Skeletal editing enables precise atomic replacement [29], deletion [30], or insertion [31] within skeleton level [32]. Similarly, molecule editing via non‐classical bioisosteres replacement allows precise structural modifications of drug, allowing for controlled modulation of physicochemical properties and dynamic behavior while preserving biological activity [33, 34]. Inspired by these frontier strategies, we break through the traditional design logic and propose a novel lattice‐editing strategy for multicomponent crystals (Scheme 1C). Different from the conventional “numerical equivalence” substitution, this strategy takes the composite local structural unit as the basic operation object and realizes precise lattice reconstruction via non‐classical bioisosteres replacement under the guidance of local‐environment equivalence (geometric compatibility, electrostatic matching, interaction topology consistency).

As a proof of concept, we applied this strategy to the rational modification of a representative ternary energetic crystal, ethylenediamine diperchlorate hemihydrate (EP·0.5H_2_O), which suffers from poor thermal stability due to lattice water. To solve this problem, we screened [H_2_pda]^2+^ as the ideal non‐classical bioisostere of the [H_2_eda]^2+^–H_2_O unit and successfully achieved the 1→2 component substitution without lattice destruction for the targeted time. The obtained anhydrous crystal PEP maintains the original high density and shows greatly improved thermal stability and formation enthalpy. More notably, PEP exhibits record‐high specific impulse in various propellant systems, significantly superior to traditional oxidizers and high‐energy oxidizer ADN. This work not only validates the feasibility of the proposed lattice‐editing strategy, but also establishes a new design paradigm for multicomponent crystal engineering that can be widely applied to diverse systems. Fundamentally, this paradigm broadens the design scope of multicomponent crystal engineering and provides an efficient and universal solution to the long‐standing key challenges in crystal structure prediction (CSP).

## 2 Results and Discussion

### 2.1 Benefits and Limitations of High‐Energy Oxidizer EP·0.5H_2_O

**FIGURE 1:**
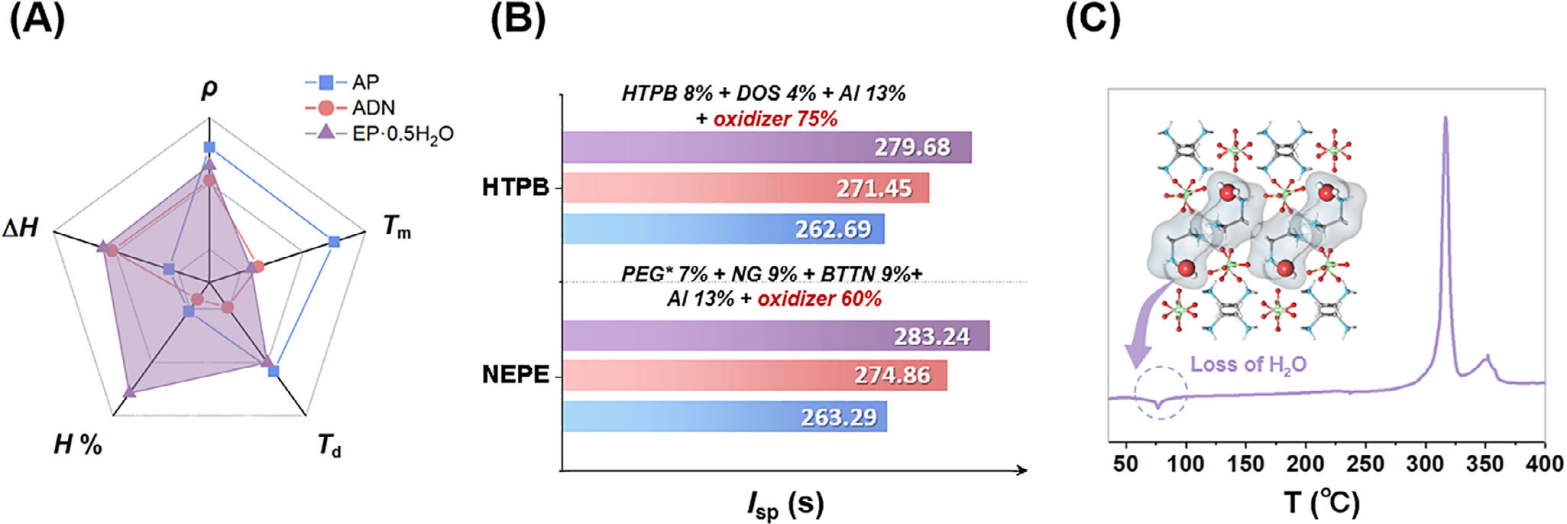
Performance characterization of EP·0.5H_2_O as an oxidizer. (A) A comparison of the performance of EP·0.5H_2_O and AP/ADN. The detailed scale ranges are: ρ (1.5–2.0 g cm^−3^), *T*_m_ (0–300°C), *T*_d_ (100–400°C), H (3–5 wt%), and Δ*H*_f_ (−400–0 kJ mol^−1^). (B) *I*_sp_ of propellant formulations employing EP·0.5H_2_O, ADN and AP as oxidizers. (C) DSC curve of EP·0.5H_2_O.

EP·0.5H_2_O shows considerable promise as a solid‐propellant oxidizer, owing to its high formation enthalpy (‐127.56 kJ mol^−1^), substantial hydrogen content (4.3%), and favorable crystal density (1.85 g cm^−3^) (Figure 1A; Table S1). Performance evaluation using the NASA CEA (Chemical Equilibrium with Applications) program indicates that formulations containing EP·0.5H_2_O deliver *I*_sp_ values of 279.68 s (Hydroxyl‐terminated polybutadiene Propellant, HTPB) and 283.24 s (Nitrate ester Plasticized Polyether Propellant, NEPE), significantly exceed those obtained with conventional ammonium perchlorate (AP) and the high‑energy oxidizer ammonium dinitramide (ADN) (Figure 1B). However, the presence of crystallization water markedly compromises its thermal stability; EP·0.5H_2_O undergoes a dehydration‐induced phase transition near 72°C (Figure 1C), which severely restricts its practical utility. Crystallographic analysis reveals a layered structure composed of alternating EP and EP·H_2_O sheets. Targeted removal of water from the EP·H_2_O layers thus offers a viable route to improve the thermal stability of EP·0.5H_2_O while retaining the advantageous energetic properties.

### 2.2 Screening of Lattice Non‐Classical Bioisostere

**SCHEME 2:**
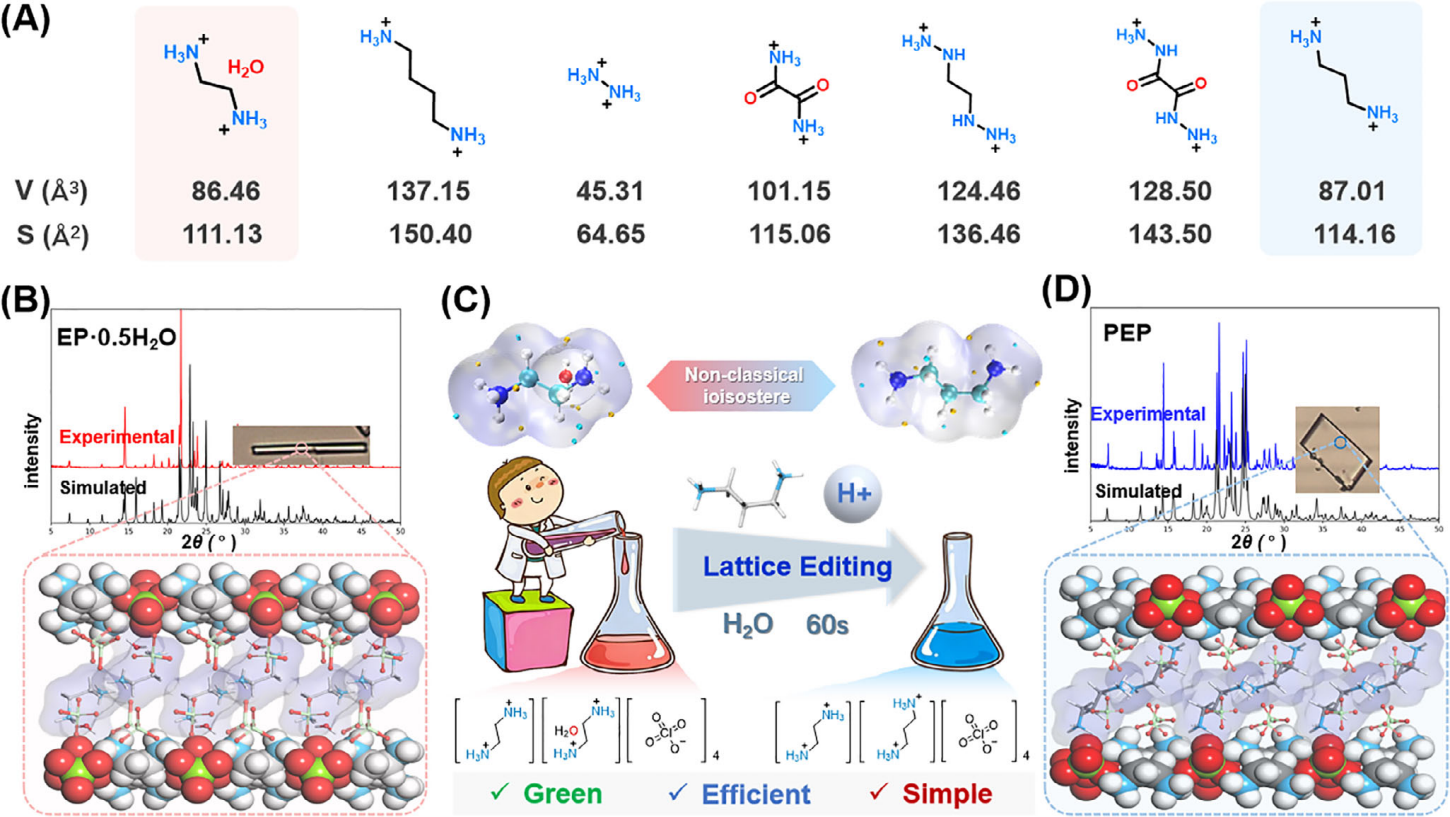
Molecular screening and crystallographic validation of the 1→2 lattice‐editing substitution. (A) Calculated volumes and surface areas of the screened diamine‐type molecules. (B) PXRD patterns and crystal packing of EP·0.5H_2_O. (C) The lattice editing reaction from EP·0.5H_2_O to PEP. (D) PXRD patterns and crystal packing of PEP.

As shown in Scheme 2B, the EP·H_2_O layers are built from [H_2_eda]^2+^ cations, perchlorate anions (ClO_4_^−^), and water molecules in a 1:2:1 stoichiometry. By treating the [H_2_eda]^2+^‐H_2_O pair as an integral structural unit, we hypothesized that its replacement with a suitable non‐classical bioisostere could eliminate water while preserving the overall lattice framework. Successful implementation requires a non‐classical bioisostere that matches the charge, hydrogen‑bonding topology, and geometric dimensions of the original [H_2_eda]^2+^‐H_2_O unit. Thus, we evaluated a series of diamine‑based cations with analogous charge and hydrogen‑bonding motifs by calculating their molecular volumes and surface areas (Scheme 2A). Among these, 1,3‐propanediammonium cation ([H_2_pda]^2+^) emerged as the most promising candidate due to its optimal geometric compatibility, as reflected in their closely matched molecular volume (86.46 Å^3^ vs 87.01 Å^3^) and surface area (113.13 Å^2^ vs 114.16 Å^2^). Moreover, the spatial distribution and intensity of the maximum electrostatic potential points of [H_2_pda]^2+^ show a high similarity to those of the [H_2_eda]^2+^‐H_2_O unit (Scheme 2C), indicating their potential interchangeability in the weak interaction network.

### 2.3 Synthesis and Structure Analysis

**FIGURE 2:**
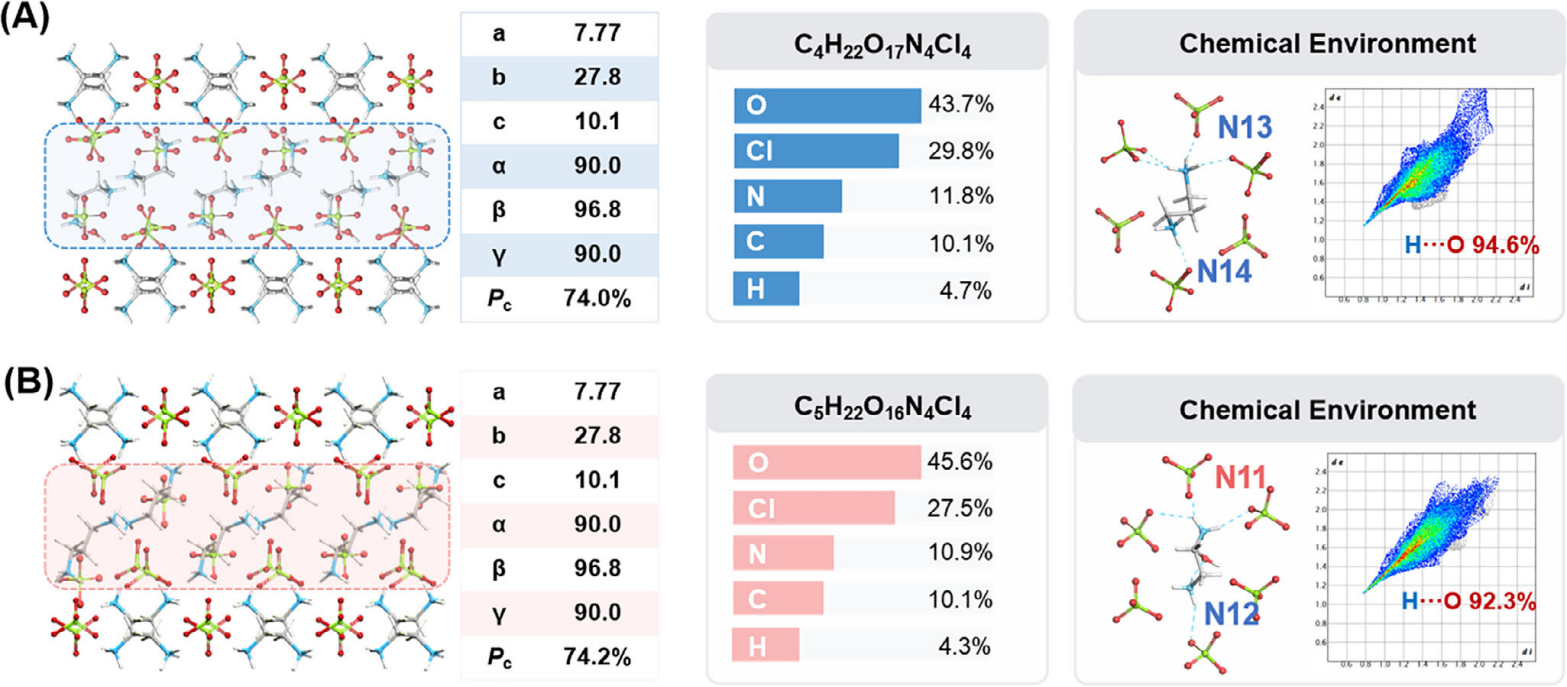
Structural and environmental comparison of EP·0.5H_2_O and PEP. (A) Crystal stacking, chemical composition, chemical environment, and weak crystal interactions of EP·0.5H_2_O. (B) Crystal stacking, chemical composition, chemical environment, and weak crystal interactions of PEP.

Following the design principle, an aqueous solution of EP·0.5H_2_O was treated with stoichiometric 1,3‑propanediamine and subsequently protonated with excess perchloric acid. The substitution proceeded smoothly in water at ambient temperature without external heating and reached completion within 60 s (Video S1), offering a safe, efficient, and green synthetic route (Scheme 2C). The product crystallized as block‐shaped crystals (78% yield), distinct from the rod‐shaped habit of the parent EP·0.5H_2_O (Scheme 2D). The experimental PXRD pattern agrees well with the simulated pattern, confirming the high phase purity of PEP. phase. Single‐crystal X‐ray diffraction analysis unambiguously revealed that the [H_2_eda]^2+^‐H_2_O units in the EP·H_2_O layers were successfully replaced by [H_2_pda]^2+^ cations, affording the non‐hydrated crystal, PEP. To our knowledge, PEP represents a previously unachieved class of ABX_4_‐type self‐assembled energetic material prepared via lattice editing. Both original [H_2_eda]^2+^‐H_2_O unit and the introduced [H_2_pda]^2+^ cation reside in highly similar chemical environments, stabilized by extensive N–H···O hydrogen‐bonding networks with surrounding perchlorate anions. Hirshfeld surface analysis confirms that N–H···O contacts formed by the ammonium groups dominate the weak interactions in both systems (94.6% and 92.3%, respectively)(Figure 2). Consequently, EP·0.5H_2_O and PEP exhibit nearly identical crystal‐packing patterns (Figures S4–S6) and comparable lattice energies (Figures S8 and S9), along with identical unit cell parameters, space groups, and closely matched packing coefficients (74.0% vs 74.2%). These results demonstrate that the substitution precisely replaces both spatial constraints and interaction networks within the lattice, achieving the targeted removal of water molecules via lattice editing.

### 2.4 Performance Analysis

Lattice‐editing preserves both the original crystal packing and the essential chemical nature of the framework. Owing to the nearly identical molecular weights of the precursor and product units (difference of only 2 amu), PEP retains a high density of 1.85 g cm^−3^, matching that of EP·0.5H_2_O (1.85 g cm^−3^). Attributed to the removal of crystal water, PEP exhibits a phase‑transition temperature of 162°C, approximately 90°C higher than that of the hydrated precursor (72°C). Furthermore, PEP displays a markedly increased molar enthalpy of formation (+35.87 kJ mol^−1^) compared with EP·0.5H_2_O. This enhancement arises primarily from the incorporation of the [H_2_pda]^2+^ cation, derived from propanediamine diperchlorate, a compound with intrinsically high enthalpy of formation (156.15 kJ mol^−1^). The elimination of crystalline water also contributes to the enhanced enthalpy of formation.

**FIGURE 3:**
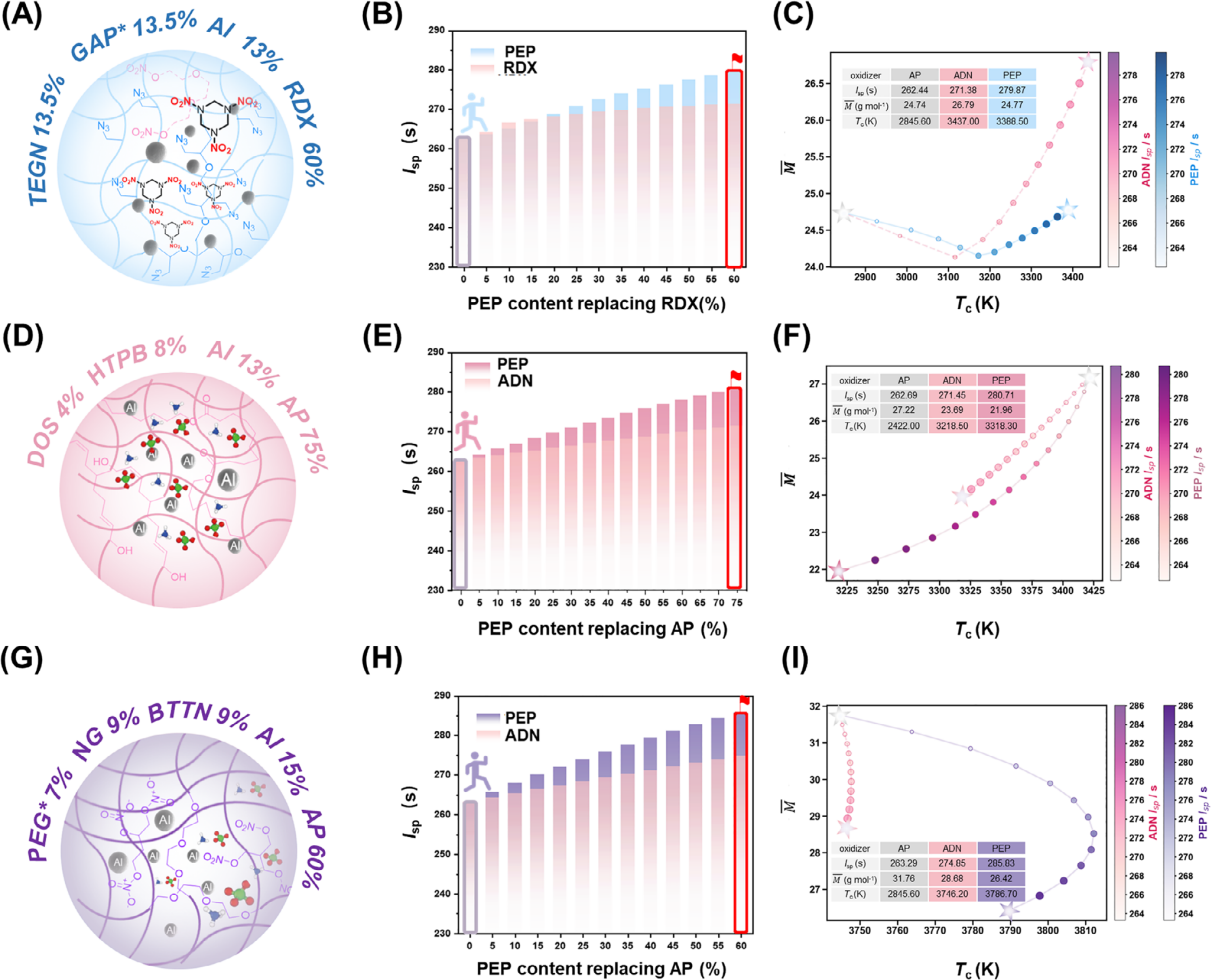
Formulation compositions and propulsion performance of propellants using PEP and ADN/AP as oxidizer substitutes. (A,D,G) The composition of composite propellant formulations. (B,E,H) Effect of PEP/ADN substitution for the oxidizer on *I*_sp_. (C,F,I) Performances of PEP and ADN replacing the oxidizer.

To evaluate the performance of PEP in solid propellants, three representative formulations‐based on glycidyl azide polymer (GAP), NEPE and HTPB‐were selected. Conventional energetic components in these formulations were systematically replaced with PEP or ADN, and the resulting performance was calculated using the NASA CEA program. As illustrated in Figure  3, PEP delivers *I*_sp_ values exceeding 280 s in all three formulations, corresponding to an increase of 18–23 s over AP‑based systems and 9–11 s over ADN‑containing formulations, underscoring its superior energetic performance. The characteristic velocity *C* ^∗^ of all three formulations is improved, indicating that PEP significantly enhances the energy release efficiency of the formulations (Tables S6–S9). The *I*_sp_ generally scales with higher chamber temperature (*T*_c_) and lower average molecular weight ($\bar{M}$) of the combustion products. The dominant mechanism for enhanced *I*_sp_ varies by formulation.

In the GAP‑based formulation (TEGN 13.5% + GAP 13.5% + Al 13%+ RDX 60%), replacing 60% of the RDX with PEP raises *T*_c_ to 3388.50 K, largely owing to the high heat of formation of PEP. Normalized contribution analysis confirms that the temperature term (*T*/*T*_0_)^1/2^ correlates strongly with the observed increase in Isp, confirming a primarily temperature‑driven improvement. With a maximum *T*_c_ of 3388.50 K—approximately 600 K higher than the AP‐based reference (2845.60 K)—the PEP‑modified formulation achieves an *I*_sp_ of 279.87 s, outperforming the ADN‑based formulation (271.38 s) due to its $\bar{M}$. In the HTPB‑based system (DOS 4% + HTPB 8% + Al 15%+ AP 75%), substituting 75% of AP with PEP creates a fuel‑rich mixture, which sharply increases the mole fraction of low‑molecular‑weight CO from 20.35% to 42.56% (Figure S12) and thereby significantly lowers the $\bar{M}$ of exhaust ($\bar{M}$_PEP_ = 21.96, $\bar{M}$_ADN_ = 23.96, $\bar{M}$_AP_ = 27.22). Although the *T*_c_ of the PEP system (3218.50 K) is slightly lower than that of the ADN system (3318.30 K), the pronounced decrease in $\bar{M}$ more than compensates for the temperature difference, yielding a higher *I*_sp_ of 280.71 s (vs. 271.45 s for ADN). The NEPE‑based formulation (PEG 7% + BTTN 9% + Al 15%+ AP 60%) exhibits a synergistic effect between elevated *T*_c_ (AP: 3744.6 K; ADN: 3746.2 K; PEP:3786.70 K) and reduced $\bar{M}$ (AP: 31.76; ADN: 28.68; PEP: 26.42) after substitution. Thus, the NEPE system achieves the highest *I*_sp_ among the three, reaching 285.83 s. In summary, the enhanced *I*_sp_ of PEP‐based formulation stems primarily increased *T*_c_ in the GAP system, reduced $\bar{M}$ in the HTPB system, and a synergistic combination in the NEPE system.

Across all three propellant systems evaluated, PEP demonstrates superior *I*_sp_ performance (279.87–285.83 s), surpassing not only the conventional oxidizer AP or RDX but also the high‐energy benchmark material ADN. To quantify this advantage, a systematic comparison of key properties between ADN and PEP was conducted, as summarized in Table 1. PEP exhibits a higher crystal density (1.85 g cm^−3^) than ADN (1.81 g cm^−3^). Furthermore, its thermal stability is significantly enhanced (Figure S17A), with both a higher phase‐transition temperature (162°C) and onset decomposition temperature (270°C) compared to ADN (147°C). In terms of environmental stability, PEP displays a higher critical relative humidity (68%) than ADN (54%), confirming its better anti‐hygroscopicity (Figure S17B). Additionally, PEP shows higher thresholds for friction sensitivity (84 N) and impact sensitivity (5 J) relative to ADN (64 N and 4 J, respectively), reflecting lower sensitivity to mechanical stimuli. These results establish the comprehensive performance advantages of PEP over ADN (Figure S17B). Importantly, PEP also offers practical benefits such as straightforward synthesis, low‐cost raw materials, and scalability, which further strengthen its potential for practical application. Consequently, PEP emerges as a highly promising energetic component for next‐generation high‐energy solid propellants.

**TABLE 1:** Properties of the ADN and the PEP.

Sample	ρ^a^ (g cm^−3^)	*T*_d_ ^b^ (°C)	CRH^c^ (%)	FS^d^ (N)	IS^e^ (J)	*I*_sp_ (s)		
						GAP^f^	HTPB^g^	NEPE^h^
ADN	1.81	147	54	64	4	271.38	271.45	274.85
PEP	1.85	270	68	84	5	279.87	280.71	285.83
EP·0.5H_2_O	1.85	276	40	84	3	281.29	279.68	283.24

^a^ Crystal density at room temperature.

^b^ Onset decomposition temperature obtained at 10 K min^−1^ for the samples.

^c^ Critical Relative Humidity.

^d^ Friction sensitivity.

^e^ Impact sensitivity.

^f^ The GAP‑based formulation: TEGN 13.5% + GAP 13.5% + Al 13%+ RDX 60%.

^g^ The HTPB‑based system: DOS 4% + HTPB 8% + Al 15%+ AP 75%.

^h^ The NEPE‑based formulation: PEG 7% + BTTN 9% + Al 15%+ AP 60%.

## 3 Conclusion

In summary, we have pioneered a lattice‐editing strategy driven by non‐classical bioisosteres replacement, which redefines the design paradigm of multicomponent crystal engineering from traditional “numerical equivalence” to innovative “local‐environment equivalence”. This breakthrough breaks the long‐term limitation of 1→1 substitution and realizes the targeted 1→2 component substitution in multicomponent crystals by identifying the [H_2_pda]^2+^ cation as a valid non‐classical bioisostere for the [H_2_eda]^2+^‐H_2_O unit. The as‐designed energetic crystal PEP maintains a high density of 1.85 g cm^−3^, with ≈90°C increase in phase‐transition temperature and 127% enhancement in formation enthalpy compared with parent crystal EP·0.5H_2_O. Benefiting from these merits, PEP delivers exceptional *I*_sp_ (279.87–285.83 s) in GAP, HTPB, and NEPE propellant formulations, outperforming AP by 17–23 s and ADN by 9–11 s, together with improved thermal stability, anti‐hygroscopicity, and mechanical safety, showing great application potential as a next‐generation high‐performance solid propellant oxidizer.

This work extends the concept of precise editing from molecular skeleton to crystalline lattice, establishing a universal design principle for rational construction of multicomponent functional crystals. By adopting composite local structural unit as the design core, the strategy extends the minimal editable structural unit in multicomponent crystals from individual components to local supramolecular structural units defined by spatial occupancy and interaction topology. This approach provides a high‐efficiency, low‐cost, and universal route for CSP, effectively addressing the critical bottlenecks of high computational cost and low accuracy in traditional CSP methods. The lattice‐editing strategy is applicable to a wide range of multicomponent systems, including pharmaceutical cocrystals, metal‐organic frameworks (MOFs), covalent organic frameworks (COFs), and hybrid perovskites, providing a new methodology for precise property regulation and performance breakthrough of advanced functional materials.

## Author Contributions

**Qinghua Zhang** and **Honglei Xia** conceived and supervised the project. **Zheng Lin**, **Min Li**, and **Honglei Xia** collected the data and developed the related codes. Zheng Lin, **Ying Wang**, **Ruihui Wang**, **Xiaoyu Feng**, **Mingjing Tang**, **Yuan Zhang**, and **Min Li** performed the data analysis. Zheng Lin, Honglei Xia, and Qinghua Zhang prepared the manuscript. All authors discussed the results and approved the final version of the manuscript.

## Conflicts of Interest

The authors declare no conflicts of interest.

## Supporting information




**Supporting File 1**: advs77573‐sup‐0001‐SuppMat.docx.

**Supporting File 2**: advs77573‐sup‐0002‐MovieS1.mp4.

## Data Availability

The data that support the findings of this study are available within the article and its Supporting Information. Additional data are available from the corresponding author upon reasonable request.
