# Supplementary material for: Redefining the Minimal Editable Unit of Supramolecular Synthons: A 1→2 Lattice‐Editing Strategy in Ternary Multicomponent Crystals
Source: Adv Sci (Weinh). 2026 Sep 3:e77573. Online ahead of print. doi: 10.1002/advs.77573 (PMC13542403; doi:10.1002/advs.77573)
Supplement: Supplementary file 1 [file ADVS-9999-e77573-s002.docx]

Supporting Information

**Redefining the Minimal Editable Unit of Supramolecular Synthons: A 1→2 Lattice-Editing Strategy in Ternary Multicomponent Crystals**

Zheng Lin,^[a,b]^ Honglei Xia,*^[a,b]^ Min Li,^[a,b]^ Ruihui Wang,^[a,c]^ Ying Wang,^[a,b]^ Xiaoyu Feng,^[a,b]^ Mingjing Tang,^[a,c]^ Yuan Zhang,^[a,c]^ and Qinghua Zhang*^[a,b]^

[a]	Prof. H. Xia, Prof. Q. Zhang, Z. Lin, M. Li, Y. Wang, X. Feng, M. Tang, Y. Zhang
School of Astronautics
Northwestern Polytechnical University
Xi’an 710065, Shaanxi, China
E-mail: xia_honglei@nwpu.edu.cn, qinghuazhang@nwpu.edu.cn.

[b]	Z. Lin, Prof. H. Xia, M. Li, Y. Wang, X. Feng, M. Tang, Y. Zhang, Prof. Q. Zhang
National Key Laboratory of Solid Propulsion
Northwestern Polytechnical University
Xi’an 710065, Shaanxi, China

[c]	R. Wang, Y. Zhang
School of Chemistry and Chemical Engineering
Northwestern Polytechnical University
Xi’an 710065, Shaanxi, China

**Table of contents**

**Figure S1.** Chemical structures and electrostatic potential (ESP) surface maps of candidate cationic building blocks.

**Figure S2.** The synthesis of **PEP**.

**Figure S3.** The synthesis of **PEP**.Asymmetric unit of **PEP**. Anisotropic displacement parameters are drawn at 50% probability.

**Figure S4.** The crystal stacking structure of **PE·0.5H_2_O** and **PEP** seen from a axis**.**

**Figure S5.** The crystal stacking structure of **PE·0.5H_2_O** and **PEP** seen from b axis.

**Figure S6.** The crystal stacking structure of **PE·0.5H_2_O** and **PEP** seen from caxis.

**Table S1.** Crystallographic data and structure refinements for **PEP** and **EP.**

**Table S2.** Selected bond distances (Å) and bond angles (°) for **PEP**.

**Table S3.** Selected hydrogen-bond geometries (Å, °) for for **PEP**.

**Table S4.** Calculated lattice energies of **EP·0.5H_2_O** and **PEP**.

**Figure S6.** DSC curves of the four samples with varied heating rates.

**Figure S7.** The infrared spectrum of **PEP**.

**Figure S8.** Calculated lattice energies of **EP·0.5H_2_O** and **PEP**.

**Figure S9.** Hirshfeld surface analysis and intermolecular interaction fingerprint plots of the lattice-isosteric building units.

**Table S5.** Thermochemical Data of Propellant Components.

**Table S6.** Propellant performance of GAP-based formulations upon substitution of **RDX** by **PEP**.

**Table S7.** Propellant performance of GAP-based formulations upon substitution of **RDX** by **ADN**.

**Table S8.** Propellant performance of HTPB-based formulations upon substitution of **AP** by **PEP**.

**Table S9.** Propellant performance of HTPB-based formulations upon substitution of **AP** by **ADN**.

**Table S10.** Propellant performance of NEPE-based formulations upon substitution of **AP** by **ADN**.

**Table S11.** Propellant performance of NEPE-based formulations upon substitution of **AP** by **PEP**.

**Figure S10.** Gas products from GAP formulations with different **ADN** and **PEP** contents.

**Figure S11.** Gas products from NEPE formulations with different **PEP** contents.

**Figure S12.** Gas products from HTPB formulations with different **PEP** contents.

**Figure S13.** Comparison of experimental and simulated PXRD patterns of **PEP** and **EP·0.5H_2_O**.

**Figure S14.** Calculated filling coefficient (or packing coefficient) of **PEP**.

**Figure S15.** Calculated filling coefficient (or packing coefficient) of **EP·0.5H_2_O**.

**Table S12.** Detonation performance of **AP, AND,** **EP·0.5H_2_O and PEP**

**Figure S16.** Thermogravimetric (TG) curve of **PEP**.

**Figure S17.** Comparative performance analysis of PEP and **ADN**.

**Figure S18.** TG–DSC curves of (A) ADN, (B) EP·0.5H_2_O, and (C) AP. The onset decomposition temperatures and the peak temperatures from DSC curves are marked.

**General Information**

All the materials and chemical reagents were analytical grade, purchased from Shanghai Aladdin Biochemical Technology Co., Ltd. and used without further purification. Infrared spectra (IR) are recorded on a Bruker Equinox 55 infrared spectrometer with KBr pellets from 400 to 4000 cm^-1^ with a resolution of 4 cm^-1^. Powder X-ray diffraction (PXRD) measurements were performed on a Bruker D8 advance diffractometer at 60 kV, 300 mA and Cu Kα radiation (*λ* = 1.5406 Å) with a scan speed of 8° min^-1^ and a step size of 0.15° in 2θ. The single-crystal X-ray diffraction data collections were carried out on a Rigaku AFC-10/Saturn 724+CCD diffractometer with graphite-monochromated Mo-Kɑ radiation (*λ* = 0.71073 Å) using a multi-scan technique. The crystal structure was determined by direct methods using SHELXS-97 and refined by full-matrix least-squares procedures on with SHELXL-97. All non-hydrogen atoms were obtained from the difference Fourier map and subjected to anisotropic refinement by full-matrix least squares on Hydrogen atoms were obtained geometrically and treated as riding on the parent atoms or were constrained in the locations during refinements. The thermal decomposition temperatures were determined by using differential scanning calorimetry (DSC) on a CDR-4 of Shanghai Precision & Scientific Instrument Co., Ltd. at heating rate of 10 ℃ min^-1^ from 40 ℃ to 350 ℃ under a dry oxygen-free nitrogen atmosphere with a flowing rate of 20 mL min^-1^. Impact and friction sensitivity measurements were made using a standard BAM Fall hammer and a BAM friction tester.

**Screening of Candidate Lattice Isosteres**

ESP is mapped onto van der Waals surfaces. The numerical values listed below each molecular structure correspond to the van der Waals volume (top row, Å^3^) and van der Waals surface area (bottom row, Å^2^). A comparative analysis reveals that the spatial volume (87.01 Å^3^) and surface area (114.16 Å^2^) of the 1,3-propanediamine cation (H_2_pda^2+^, top middle) are highly congruent with the parameters of the target "ethylenediammonium–water" dual-component unit (H2eda^2+^+H_2_O, top right; 86.46 Å^3^ and 111.13 Å^2^). This exceptional similarity in spatial occupancy and charge distribution validates the structural feasibility of employing H_2_pda^2+^ as a lattice isostere to achieve 1-to-2 substitution.

**Figure S1.** Chemical structures and electrostatic potential (ESP) surface maps of candidate cationic building blocks

**Synthesis of EP·0.5H_2_O**

Ethylenediamine was dissolved in 5 mL of water at room temperature through vigorous stirring (600 rpm). followed by the addition of 4 mmol of perchloric acid solution. The resulting precipitate was filtered and washed with anhydrous ethanol (2×3 mL), then dried under sunlight and subsequently under vacuum to obtain the target compound **EP·0.5H_2_O** as a white solid.

**Synthesis of PEP**

**EP·0.5H_2_O** (0.54 g, 1 mmol) was dissolved in 5 mL of water at room temperature through vigorous stirring (600 rpm). Then, 1,3-diaminopropane (1 mmol) was added to the above mixture and stirred for 2 minutes, followed by the addition of 4 mmol of perchloric acid solution. The reaction mixture became clear and colorless within 60 seconds. The resulting precipitate was filtered and washed with anhydrous ethanol (2×3 mL), then dried under sunlight and subsequently under vacuum to obtain the target compound **PEP** as a colorless solid. Yield: 0.413 g, 77.1%. DSC (10 °C min−1, °C): 161.3 (dec.); IR (KBr pellet, cm−1): ṽ 2985, 1592, 1510, 1464, 1408, 1351, 1149, 1007，942，775，627 (s).

**Figure S2.** The synthesis of **PEP**

**Orted Drawings of the Crystal Structure**

**Figure S3.** Asymmetric unit of **PEP**. Anisotropic displacement parameters are drawn at 50% probability

**Crystallographic data**

Crystal packing viewed along the a, b, and c axes.

**Figure S4.** The crystal stacking structure of **EP·0.5H_2_O** and **PEP** seen from *a* axis

**Figure S5.** The crystal stacking structure of **EP·0.5H_2_O** and **PEP** seen from *b* axis

**Figure S6.** The crystal stacking structure of **EP·0.5H_2_O** and **PEP** seen from *c* axis

To visually underscore the conservation of crystal packing following lattice editing and to highlight the specific editing sites, background color bands (light blue) are employed to demarcate characteristic layered packing motifs. A comparison reveals that, despite undergoing a "1-to-2" chemical substitution, the novel crystal **PEP** (c) faithfully inherits the overall lattice framework and packing topology of the parent **EP·0.5H_2_O** (b), as evidenced by the similar ionic arrangement and interlayer spacing. This observation serves as direct visual validation of the efficacy of the lattice isosterism strategy in maintaining structural stability.

**Table S1.** Crystallographic data and structure refinements for **PEP** and **EP**

| Compound | **PEP** | **EP·0.5H_2_O** |
| --- | --- | --- |
| Formula | C_5_H_22_Cl_4_N_4_O_16_ | C_4_H_22_Cl_4_N_4_O_17_ |
| Formula weight | 536.06 | 540.05 |
| *T*/K | 298(0) | 293(2) |
| Crystal system | Monoclinic | Monoclinic |
| Space group | *P2_1_/c* | *P2_1_/c* |
| *a/Å* | 7.8549(10) | 7.76980(10) |
| *b/Å* | 24.742(3) | 24.7985(5) |
| *c/Å* | 9.9887(12) | 10.1106(2) |
| *α*/*°* | 90 | 90 |
| *β*/*°* | 97.606(5) | 96.803(2) |
| *γ*/*°* | 90 | 90 |
| Volume/Å^3^ | 1924.2(4) | 1934.39(6) |
| Z | 4 | 4 |
| Dc /g cm^-3^ | 1.850 | 1.854 |
| Reflns coll. | 10188 | 13851 |
| R_1_ [*I*>2σ(*I*)] | 0.0934 | 0.0439 |
| *w*R_2_ [*I*>2σ(*I*)] | 0.1971 | 0.1123 |
| R_1_ (all data) | 0.2044 | 0.0518 |
| *w*R_2_ (all data) | 0.2464 | 0.1167 |
| λ | 0.71073 Å | 1.54184 Å |
| GooF | 1.027 | 1.080 |
| F（000） | 1104 | 1112 |
| μ | 0.703 mm⁻¹ | 6.450 mm⁻¹ |
| Rint | 0.1477 | 0.0375 |
| 2θ range | 4.43–49.99 | 7.13-152.67 |
| CCDC | 2522678 | 2419070 |

**Table S2.** Selected bond distances (Å) and bond angles (°) for **PEP**

| **PEP** |  |  |  |
| --- | --- | --- | --- |
| O1-O005 | 1.442(6) | Cl02-O00J | 1.434(8) |
| Cl01-O007 | 1.449(7) | Cl02-O00K | 1.417(8) |
| Cl01-O008 | 1.446(7) | Cl03-O00B | 1.446(7) |
| Cl01-O00E | 1.441(7) | Cl03-O00D | 1.443(8) |
| Cl02-O009 | 1.435(8) | Cl02-O006 | 1.452(7) |
| Cl03-O00L | 1.411(8) | N00A-C00R | 1.482(13) |
| Cl04-O00C | 1.422(7) | N00A-C00N | 1.493(12) |
| Cl04-O00I | 1.404(9) | N00A-C00S | 1.473(14) |
| Cl04-O00P | 1.396(9) | C00N-C00O | 1.467(14) |
| Cl04-O1 | 1.402(9) | C00Q-C00R | 1.520(15) |
| N00A-C00O | 1.487(1) | C00Q-C00S | 1.475(16) |
| Cl03-O00F | 1.407(8) |  |  |
|  |  |  |  |
| O005-Cl01-O007 | 108.2(4) | O00F-Cl03-O00L | 110.8(6) |
| O005-Cl01-O008 | 109.3(4) | O00L-Cl03-O00B | 110.0(5) |
| O008-Cl01-O007 | 109.5(5) | O00L-Cl03-O00D | 110.3(6) |
| O00E-Cl01-O005 | 110.7(5) | O00I-Cl04-O00C | 108.9(5) |
| O00E-Cl01-O007 | 109.6(4) | O00P-Cl04-O00P | 108.9(5) |
| O00E-Cl01-O008 | 109.6(5) | O00P-Cl04-O00I |  |
| O009-Cl02-O006 | 108.1(5) | O00P -Cl04-O1 |  |
| O00J-Cl02-O006 | 108.7(5) | O1-Cl04-O00C | 108.9(5) |
| O00K-Cl02-O009 | 110.4(5) | O1-Cl04-O00I | 108.9(5) |
| O00K-Cl02-O006 | 109.5(5) | O00O-Cl0N-O00C | 114.4(8) |
| O00K-Cl02-O009 | 112.4(6) | O00N-Cl0O-O00C | 114.3(9) |
| O00K-Cl02-O00J | 108.8(4) | C00S-C00Q-C00R | 114.0(11) |
| O00F-Cl03-O00B | 108.7(5) | N00G-C00R-C00Q | 114.0(11) |
| O00D-Cl03-O00B | 108.8(4) | N00M-C00S-C00Q | 112.6(10) |
| O00F-Cl03-O00D | 108.2(5) | 108.2(5) |  |

**Table S3.** Selected hydrogen-bond geometries (Å, °) for for **PEP**

| **D−H⋯A** | ***d*_(D−H)_/Å** | ***d*_(H⋯A)_/Å** | ***d*_(D⋯A)_/Å** | **D−H⋯A/ °** |
| --- | --- | --- | --- | --- |
| N00A-H00M···O00C | 0.8900 | 2.5500 | 2.945(11) | 107.00 |
| N00A-H00M···O006 | 0.8900 | 2.5400 | 3.002(11) | 113.00 |
| N00A-H00M···O007 | 0.8900 | 2.2400 | 3.016(11) | 146.00 |
| N00A-H00N···O00B | 0.8900 | 2.0900 | 2.920(11) | 156.00 |
| N00A-H00N···O00D | 0.8900 | 2.5500 | 3.087(11) | 119.00 |
| N00A-H00N···O009 | 0.8900 | 2.5000 | 3.056(11) | 121.00 |
| N00A-H00O···O005 | 0.8900 | 2.2400 | 3.055(11) | 153.00 |
| N00A-H00O···O007 | 0.8900 | 2.5500 | 3.274(11) | 139.00 |
| N00G-H00A···O00E | 0.8900 | 2.5500 | 3.210(11) | 132.00 |
| N00G-H00A···O00F | 0.8900 | 2.2300 | 2.994(12) | 143.00 |
| N00G-H00C···O1 | 0.8900 | 2.2100 | 2.972(13) | 143.00 |
| N00H-H00P···O006 | 0.8900 | 2.2800 | 2.959(11) | 133.00 |
| N00H-H00P···O008 | 0.8900 | 2.4400 | 3.195(11) | 143.00 |
| N00H-H00Q···O005 | 0.8900 | 2.2600 | 3.002(11) | 141.00 |
| N00H-H00Q ···O008 | 0.8900 | 2.5000 | 3.209(11) | 137.00 |
| N00H-H00R···O00B | 0.8900 | 2.3500 | 2.938(11) | 124.00 |
| N00H-H00R···O00C | 0.8900 | 2.1200 | 2.883(11) | 143.00 |
| N00M-H00D···O009 | 0.8900 | 2.1500 | 2.957(13) | 150.00 |
| N00M-H00E···O00L | 0.8900 | 2.0900 | 2.898(15) | 151.00 |
| C00S-H00K···O009 | 0.9700 | 2.5500 | 3.431(15) | 151.00 |

**Fourier transform infrared spectroscopy (FTIR)**

**Figure S7.** The infrared spectrum of **PEP**

**Lattice Energy Calculation**

The enthalpies of formation for the individual components (*∆H*_A_、*∆H*_B_、*∆H*_X_) were calculated using the Gaussian software package at the BMK/6-31+G(2df,p) level (see Note below).Subsequently, the total energy of the periodic unit cell (*E*_total_) and the energies of the individual components within the same periodic system (*E*_A_*、E*_B_*、E*_X_) were calculated using the CP2K software package. These calculations employed the PBE functional combined with GTH pseudopotentials and the DZVP basis set. Dispersion corrections were applied using the DFT-D3(BJ) method.The lattice energy (*E*_lattice_) was determined using Equation (1):

*E*_lattice_*=E*_total_*−*(*n*_A_*E*_A_*+n*_B_*E*_B_*+n*_X_*E*_X_) (1)

Finally, the enthalpy of formation was calculated using Equation (2):

*ΔH*=*n*_A_*∆H*_A_*+n*_B_*∆H*_A_*+n*_X_*∆H*_A_−｜*E*_lattice_｜ (2)

Note: Enthalpy of formation calculated using the composite chemistry method.The enthalpy (*H*) for compound at standard condition (298K, 1atm) were directly calculated by G4(MP2)-6x method with Gaussian 09 (Revision D.01) suite of program. G4(MP2)-6x method (*J. Chem. Theory Comput., 2011, 7, 112-120*) is a composite procedure with a lower cost but performance approaching that of G4. In this method, geometries were optimized with the BMK functional using the 6-31+G(2df,p) basis set. Zero-point vibrational energies (ZPVEs) and thermal corrections to enthalpy (*ΔH*) at 298K, derived from scaled BMK/6-31+G(2df,p) frequencies, were incorporated into the total energies. Single-point energies were obtained at the HF/GFHFB3, HF/GFHFB4, MP2(FrzG4)/GTMP2LargeXP, and CCSD(T,FrzG4)/GTBas1 levels with composite procedures.

After acquiring the enthalpy of compounds, their gas phase enthalpy of formation can be calculated by atomization method as following equation (1):

$$\text{∆}\text{H}_{\text{f}}\text{(g)=}\text{H}\left( \text{C}_{\text{l}}\text{H}_{\text{m}}\text{O}_{\text{n}}\text{N}_{\text{i}}\text{(g)} \right)\text{-l}\text{H}\left( \text{C}\left( \text{g} \right) \right)\text{-}\frac{\text{m}}{\text{2}}\text{H}\left( \text{H}_{\text{2}}\left( \text{g} \right) \right)\text{-}\frac{\text{n}}{\text{2}}\text{H}\left( \text{O}_{\text{2}}\left( \text{g} \right) \right)\text{-}$$

$\frac{\text{i}}{\text{2}}\text{H}\left( \text{N}_{\text{2}}\left( \text{g} \right) \right)\text{+l}\text{H}_{\text{vap}}\text{(}\text{grap}\text{h}\text{ite}\text{)}$	(1)

For neutral compounds with calculated gas state heat of formation, the solid state heat of formation was calculated by the following equation (2). The heat of sublimation $\text{∆}\text{H}_{\text{SUB}}$ was estimated by the following equation (3). T is the melting point or decomposition point temperature in Kelvin.

$\text{∆}\text{H}_{\text{f}}\left( \text{s} \right)\text{=}\text{∆}\text{H}_{\text{f}}\left( \text{g} \right)\text{-∆}\text{H}_{\text{SUB}}$	(2)

$\text{∆}\text{H}_{\text{SUB}}\left( \text{g} \right)\text{=0.188*}\text{T}$	(3)

For ionic compound, solid state heat of formation was calculated by equation (4). In the equation, enthalpy of lattice $\text{∆}\text{H}_{\text{L}}$ was estimated by equation (5) and (6). In equation 5 and 6, p，q are charge number for cation and anion; $\text{n}_{\text{M}}$ , $\text{n}_{\text{X}}$, $\text{γ}$ and $\text{δ}$ are constant decided by structure of ion; $\text{ρ}_{\text{m}}$ and $\text{M}_{\text{m}}$ are density and molecular weight. More details about the parameters for equation 7 and 8 can be found in *Inorg. Chem. 2002, 41, 2364−2367.*

$\text{∆}\text{H}_{\text{f}}\left( \text{ion,s} \right)\text{=m∆}\text{H}_{\text{f}}\left( \text{cation,g} \right)\text{+n∆}\text{H}_{\text{f}}\left( \text{anion,g} \right)\text{-∆}\text{H}_{\text{L}}$	(4)

$\text{∆}\text{H}_{\text{L}}\text{=}\text{U}_{\text{pot}}\text{+}\left[ \text{p}\left( \frac{\text{n}_{\text{M}}}{\text{2}}\text{-2} \right)\text{+}\text{q}\left( \frac{\text{n}_{\text{X}}}{\text{2}}\text{-2} \right) \right]\text{RT}$ (5)

$\text{U}_{\text{pot}}\text{=}\text{γ}{\text{(}\frac{\text{ρ}_{\text{m}}}{\text{M}_{\text{m}}}\text{)}}^{\text{1/3}}\text{+}\text{δ}$ (6)

**Table S4.** Calculated lattice energies of **EP·0.5H_2_O** and **PEP**

| Sample | *E*_lattice_ (kJ mol^-1^) |
| --- | --- |
| **EP·0.5H_2_O** | -9660.11573 |
| **PEP** | -8914.797305 |

**Figure S8.** Calculated lattice energies of **EP·0.5H_2_O** and **PEP**

**Figure S9.** Hirshfeld surface analysis and intermolecular interaction fingerprint plots of the lattice-isosteric building units

Caption:(a, b) Hirshfeld surfaces (mapped with *d*_norm_) and corresponding 2D fingerprint plots for the 1,3-propanediamine cation (H_2_pda^2+^) in **PEP** (a) and the "ethylenediammonium–water" dual-component unit (H_2_eda^2+^·H_2_O) in the parent **EP·0.5H_2_O** (b). Red regions on the surfaces denote close contacts, primarily corresponding to strong N–H···O hydrogen bonding interactions. The characteristic spikes in the fingerprint plots and the indicated percentage values (94.2% and 94.0%) reveal that H···O contacts (with perchlorate anions) dominate the total surface interactions in both cases, with highly comparable contribution ratios. This extensive overlap of interaction profiles confirms that the lattice isostere maintains a supramolecular behavior highly consistent with that of the parent unit.

**Solid Propellant Applications**

Distribution of equilibrium combustion products for solid propellants as a function of **PEP** content.

**Table S5.** Thermochemical Data of Propellant Components

| Compound | Chemical Formula | HOF  (kJ·mol⁻¹) |
| --- | --- | --- |
| Al (Aluminum) | Al 1 | 0.0 ^b^ |
| HTPB (Hydroxyl-terminated polybutadiene) | C 10.00 H 15.40 O 0.070 | −51.88 ^a^ |
| AP (Ammonium perchlorate) | N 1 H 4 Cl 1 O 4 | −295.77 ^b^ |
| GAP (Glycidyl azide polymer) | C 3 H 5 N 3 O 1 | +138.0 ^a^ |
| NG (Nitroglycerin) | C 3 H 5 O 9 N 3 | −372.5 ^a^ |
| PEG (Polyethylene glycol) | C 45 H 91 O 23 | −442.8 ^a^ |
| ADN (Ammonium dinitramide) | N 4 H 4 O 4 | −150.0 ^a^ |
| BTTN (1,2,4-Butanetriol trinitrate) | C 4 H 7 O 9 N 3 | −406.98 ^a^ |
| Data were obtained from published literature sources. Data were obtained from the NASA CEA database. | | |

The performance parameters, including specific impulse (*I*_sp_), characteristic velocity (C*), combustion chamber temperature (*T*_c_), and equilibrium gas products, were calculated for three classic propellant formulations—Glycidyl Azide Polymer (GAP), Hydroxyl-terminated Polybutadiene (HTPB), and Nitrate Ester Plasticized Polyether (NEPE)—using the NASA CEA software at an operating pressure of 0.1 MPa. Specifically, the GAP-based formulation consisted of 13 wt% GAP, 13 wt% Triethylene Glycol Dinitrate (**TEGN**), 13 wt% Aluminum (**Al**), and a balance of oxidizers. The oxidizer system was composed of Cyclotrimethylenetrinitramine (**RDX**) and **PEP**. Calculations were performed by varying the **RDX** content from 0% to 60% (with **PEP** adjusted accordingly) in increments of 5%.

The NEPE-based formulation consisted of 9 wt% Nitroglycerin (**NG**), 7 wt% Polyethylene Glycol (**PEG**), 9 wt% 1,2,4-Butanetriol Trinitrate (**BTTN**), 15 wt% Aluminum (**Al**), and a balance of oxidizers. The oxidizer system was composed of Ammonium Perchlorate (**AP**) and **PEP**. Calculations were performed by varying the **AP** mass fraction from 0% to 60% (with the **PEP** content adjusted accordingly) in increments of 5%.

The HTPB-based formulation consisted of 8 wt% Hydroxyl-terminated Polybutadiene (HTPB), 4 wt% Dioctyl Sebacate (**DOS**), 13 wt% Aluminum (**Al**), and 75 wt% oxidizers. The oxidizer system was composed of Ammonium Perchlorate (**AP**) and **PEP**. Calculations were performed by varying the **AP** mass fraction from 0% to 75% (with the **PEP** content adjusted accordingly) in increments of 5%.

**Table S6.** Propellant performance of GAP-based formulations upon substitution of **RDX** by **PEP**

| **TEGN** | **GAP** | **Al** | **RDX** | **PEP** | *I*_sp_ (s) | *T*_c_ (K) | C*  (m s^-1^) | $\bar{M}$  (g mol^-1^) |
| --- | --- | --- | --- | --- | --- | --- | --- | --- |
| 13.5 | 13.5 | 13 | 0 | 60 | 279.87 | 3388.5 | 1693.7 | 24.77 |
| 13.5 | 13.5 | 13 | 5 | 55 | 278.8 | 3363.1 | 1688.2 | 24.68 |
| 13.5 | 13.5 | 13 | 10 | 50 | 277.68 | 3336.4 | 1682.4 | 24.58 |
| 13.5 | 13.5 | 13 | 15 | 45 | 276.52 | 3308.2 | 1676.2 | 24.49 |
| 13.5 | 13.5 | 13 | 20 | 40 | 275.32 | 3278.3 | 1669.6 | 24.39 |
| 13.5 | 13.5 | 13 | 25 | 35 | 274.07 | 3246.2 | 1662.3 | 24.3 |
| 13.5 | 13.5 | 13 | 30 | 30 | 272.71 | 3211.3 | 1654.1 | 24.2 |
| 13.5 | 13.5 | 13 | 35 | 25 | 271 | 3172.7 | 1644.6 | 24.15 |
| 13.5 | 13.5 | 13 | 40 | 20 | 268.87 | 3128.6 | 1633.2 | 24.26 |
| 13.5 | 13.5 | 13 | 45 | 15 | 266.86 | 3076.3 | 1619.3 | 24.38 |
| 13.5 | 13.5 | 13 | 50 | 10 | 265.07 | 3010.3 | 1601.5 | 24.5 |
| 13.5 | 13.5 | 13 | 55 | 5 | 263.58 | 2927.7 | 1579.7 | 24.62 |
| 13.5 | 13.5 | 13 | 60 | 0 | 262.44 | 2845.6 | 1579.8 | 24.74 |

**Table S7.** Propellant performance of GAP-based formulations upon substitution of **RDX** by **ADN**

| **TEGN** | **GAP** | **Al** | **RDX** | **ADN** | *I*_sp_ (s) | *T*_c_ (K) | C*  (m s^-1^) | $\bar{M}$  (g mol^-1^) |
| --- | --- | --- | --- | --- | --- | --- | --- | --- |
| 13.5 | 13.5 | 13 | 0 | 60 | 271.38 | 3437 | 1636.7 | 26.79 |
| 13.5 | 13.5 | 13 | 5 | 55 | 271.18 | 3416.4 | 1637.4 | 26.5 |
| 13.5 | 13.5 | 13 | 10 | 50 | 270.92 | 3394 | 1637.7 | 26.21 |
| 13.5 | 13.5 | 13 | 15 | 45 | 270.6 | 3369.8 | 1637.5 | 25.93 |
| 13.5 | 13.5 | 13 | 20 | 40 | 270.23 | 3344 | 1637 | 25.66 |
| 13.5 | 13.5 | 13 | 25 | 35 | 269.8 | 3316.6 | 1636.1 | 25.39 |
| 13.5 | 13.5 | 13 | 30 | 30 | 269.31 | 3287.6 | 1634.9 | 25.13 |
| 13.5 | 13.5 | 13 | 35 | 25 | 268.77 | 3256.7 | 1633.3 | 24.87 |
| 13.5 | 13.5 | 13 | 40 | 20 | 268.15 | 3223.1 | 1631.3 | 24.61 |
| 13.5 | 13.5 | 13 | 45 | 15 | 267.48 | 3183.2 | 1628.9 | 24.37 |
| 13.5 | 13.5 | 13 | 50 | 10 | 266.53 | 3116.2 | 1621.9 | 24.13 |
| 13.5 | 13.5 | 13 | 55 | 5 | 264.19 | 2983.7 | 1589.4 | 24.42 |
| 13.5 | 13.5 | 13 | 60 | 0 | 262.44 | 2845.6 | 1579.8 | 24.74 |

**Table S8.** Propellant performance of HTPB-based formulations upon substitution of **AP** by **PEP**

| **HTPB** | **DOS** | **Al** | **AP** | **PEP** | *I*_sp_ (s) | *T*_c_ (K) | C*  (m s^-1^) | $\bar{M}$  (g mol^-1^) |
| --- | --- | --- | --- | --- | --- | --- | --- | --- |
| 13.5 | 13.5 | 13 | 0 | 60 | 279.87 | 3388.5 | 1693.7 | 24.77 |
| 13.5 | 13.5 | 13 | 5 | 55 | 278.8 | 3363.1 | 1688.2 | 24.68 |
| 13.5 | 13.5 | 13 | 10 | 50 | 277.68 | 3336.4 | 1682.4 | 24.58 |
| 13.5 | 13.5 | 13 | 15 | 45 | 276.52 | 3308.2 | 1676.2 | 24.49 |
| 13.5 | 13.5 | 13 | 20 | 40 | 275.32 | 3278.3 | 1669.6 | 24.39 |
| 13.5 | 13.5 | 13 | 25 | 35 | 274.07 | 3246.2 | 1662.3 | 24.3 |
| 13.5 | 13.5 | 13 | 30 | 30 | 272.71 | 3211.3 | 1654.1 | 24.2 |
| 13.5 | 13.5 | 13 | 35 | 25 | 271 | 3172.7 | 1644.6 | 24.15 |
| 13.5 | 13.5 | 13 | 40 | 20 | 268.87 | 3128.6 | 1633.2 | 24.26 |
| 13.5 | 13.5 | 13 | 45 | 15 | 266.86 | 3076.3 | 1619.3 | 24.38 |
| 13.5 | 13.5 | 13 | 50 | 10 | 265.07 | 3010.3 | 1601.5 | 24.5 |
| 13.5 | 13.5 | 13 | 55 | 5 | 263.58 | 2927.7 | 1579.7 | 24.62 |
| 13.5 | 13.5 | 13 | 60 | 0 | 262.44 | 2845.6 | 1579.8 | 24.74 |

**Table S9.** Propellant performance of HTPB-based formulations upon substitution of **AP** by **ADN**

| **HTPB** | **DOS** | **Al** | **AP** | **ADN** | *I*_sp_ (s) | *T*_c_ (K) | C*  (m s^-1^) | $\bar{M}$  (g mol^-1^) |
| --- | --- | --- | --- | --- | --- | --- | --- | --- |
| 8 | 4 | 13 | 0 | 75 | 271.45 | 3318.3 | 1640.6 | 23.96 |
| 8 | 4 | 13 | 5 | 70 | 270.93 | 3325.8 | 1636.8 | 24.16 |
| 8 | 4 | 13 | 10 | 65 | 270.4 | 3333.2 | 1632.9 | 24.35 |
| 8 | 4 | 13 | 15 | 60 | 269.87 | 3340.5 | 1629 | 24.55 |
| 8 | 4 | 13 | 20 | 55 | 269.33 | 3347.7 | 1625 | 24.75 |
| 8 | 4 | 13 | 25 | 50 | 268.77 | 3354.9 | 1620.9 | 24.96 |
| 8 | 4 | 13 | 30 | 45 | 268.21 | 3362 | 1616.8 | 25.17 |
| 8 | 4 | 13 | 35 | 40 | 267.64 | 3369 | 1612.6 | 25.38 |
| 8 | 4 | 13 | 40 | 35 | 267.05 | 3375.9 | 1608.4 | 25.6 |
| 8 | 4 | 13 | 45 | 30 | 266.46 | 3382.7 | 1604.1 | 25.82 |
| 8 | 4 | 13 | 50 | 25 | 265.86 | 3389.5 | 1599.7 | 26.04 |
| 8 | 4 | 13 | 55 | 20 | 265.25 | 3396.2 | 1595.3 | 26.27 |
| 8 | 4 | 13 | 60 | 15 | 264.63 | 3402.7 | 1590.8 | 26.5 |
| 8 | 4 | 13 | 65 | 10 | 264 | 3409.2 | 1586.2 | 26.74 |
| 8 | 4 | 13 | 70 | 5 | 263.35 | 3415.7 | 1581.6 | 26.98 |
| 8 | 4 | 13 | 75 | 0 | 262.69 | 3422 | 1576.9 | 27.22 |

**Table S10.** Propellant performance of NEPE-based formulations upon substitution of **AP** by **ADN**

| **PEG*** | **NG** | **BTTN** | **Al** | **AP** | **ADN** | *I*_sp_ (s) | *T*_c_ (K) | C*  (m s^-1^) | $\bar{M}$  (g mol^-1^) |
| --- | --- | --- | --- | --- | --- | --- | --- | --- | --- |
| 7 | 9 | 9 | 15 | 0 | 60 | 274.85 | 3746.2 | 1633.5 | 28.68 |
| 7 | 9 | 9 | 15 | 5 | 55 | 273.97 | 3746.7 | 1627.4 | 28.93 |
| 7 | 9 | 9 | 15 | 10 | 50 | 273.06 | 3747.2 | 1621.3 | 29.18 |
| 7 | 9 | 9 | 15 | 15 | 45 | 272.14 | 3747.5 | 1615.1 | 29.43 |
| 7 | 9 | 9 | 15 | 20 | 40 | 271.21 | 3747.6 | 1608.9 | 29.68 |
| 7 | 9 | 9 | 15 | 25 | 35 | 270.27 | 3747.7 | 1602.7 | 29.94 |
| 7 | 9 | 9 | 15 | 30 | 30 | 269.31 | 3747.6 | 1596.4 | 30.20 |
| 7 | 9 | 9 | 15 | 35 | 25 | 268.34 | 3747.4 | 1590 | 30.45 |
| 7 | 9 | 9 | 15 | 40 | 20 | 267.35 | 3747 | 1583.6 | 30.71 |
| 7 | 9 | 9 | 15 | 45 | 15 | 266.35 | 3746.6 | 1577.2 | 30.97 |
| 7 | 9 | 9 | 15 | 50 | 10 | 265.34 | 3746 | 1570.7 | 31.23 |
| 7 | 9 | 9 | 15 | 55 | 5 | 264.32 | 3745.3 | 1564.2 | 31.49 |
| 7 | 9 | 9 | 15 | 60 | 0 | 263.29 | 3744.6 | 1557.7 | 31.76 |

**Table S11.** Propellant performance of NEPE-based formulations upon substitution of **AP** by **PEP**

| **PEG*** | **NG** | **BTTN** | **Al** | **AP** | **PEP** | *I*_sp_ (s) | *T*_c_ (K) | C*  (m s^-1^) | $\bar{M}$  (g mol^-1^) |
| --- | --- | --- | --- | --- | --- | --- | --- | --- | --- |
| 7 | 9 | 9 | 15 | 0 | 60 | 285.83 | 3786.7 | 1710.3 | 26.42 |
| 7 | 9 | 9 | 15 | 5 | 55 | 284.32 | 3795 | 1699.8 | 26.82 |
| 7 | 9 | 9 | 15 | 10 | 50 | 282.75 | 3801.7 | 1689 | 27.23 |
| 7 | 9 | 9 | 15 | 15 | 45 | 281.13 | 3806.5 | 1677.7 | 27.65 |
| 7 | 9 | 9 | 15 | 20 | 40 | 279.46 | 3809.5 | 1666.1 | 28.09 |
| 7 | 9 | 9 | 15 | 25 | 35 | 277.72 | 3810.4 | 1654.1 | 28.52 |
| 7 | 9 | 9 | 15 | 30 | 30 | 275.92 | 3809.2 | 1641.7 | 28.98 |
| 7 | 9 | 9 | 15 | 35 | 25 | 274.05 | 3805.7 | 1628.9 | 29.43 |
| 7 | 9 | 9 | 15 | 40 | 20 | 272.1 | 3799.6 | 1615.6 | 29.9 |
| 7 | 9 | 9 | 15 | 45 | 15 | 270.05 | 3790.8 | 1601.9 | 30.37 |
| 7 | 9 | 9 | 15 | 50 | 10 | 267.92 | 3778.9 | 1587.7 | 30.84 |
| 7 | 9 | 9 | 15 | 55 | 5 | 265.67 | 3763.6 | 1573 | 31.3 |
| 7 | 9 | 9 | 15 | 60 | 0 | 263.29 | 3744.6 | 1557.7 | 31.76 |

**Figure S10.** Gas products from GAP formulations with different **ADN** and **PEP** contents

**Figure S11.** Gas products from NEPE formulations with different **PEP** contents

**Figure S12.** Gas products from HTPB formulations with different **PEP** contents

**Figure S13.** Comparison of experimental and simulated PXRD patterns of **PEP** and **EP·0.5H_2_O**

**Filling Coefficient**

**Figure S14.** Calculated filling coefficient (or packing coefficient) of **PEP**

**Figure S15.** Calculated filling coefficient (or packing coefficient) of **EP·0.5H_2_O**

**Detonation Performance**

**Table S12.** Detonation performance of **AP, AND,** **EP·0.5H_2_O and PEP**

| Compound | ρ(g/cm^3^) | Δ*H*_f_ (kJ/mol) | IS  (J) | FS  (N) | D(m/s) | P(GPa) |
| --- | --- | --- | --- | --- | --- | --- |
| **AP** | 1.95 | -295.8 | 15 | 320 | 6809 | 18.6 |
| **ADN** | 1.81 | -150 | 3-5 | 64-72 | 8502 | 21 |
| **EP·0.5H_2_O** | 1.85 | -127.56 | 3 | 84 | 8532.64 | 34.01 |
| **PEP** | 1.85 | 35.87 | 5 | 84 | 8518.89 | 32.95 |

**Figure S16.** Thermogravimetric (TG) curve of **PEP**

**Figure S17.** Comparative performance analysis of **PEP** and **ADN**

**Figure S18.** TG–DSC curves of (A) ADN, (B) EP·0.5H_2_O, and (C) AP. The onset decomposition temperatures and the peak temperatures from DSC curves are marked.
